# Effectiveness of lower target temperature therapeutic hypothermia in post-cardiac arrest syndrome patients with a resuscitation interval of ≤30 min

**DOI:** 10.1186/s40560-015-0095-2

**Published:** 2015-06-18

**Authors:** Tadashi Kaneko, Shunji Kasaoka, Takashi Nakahara, Hirotaka Sawano, Yoshio Tahara, Mamoru Hase, Kenji Nishioka, Shinichi Shirai, Hiroshi Hazui, Hideki Arimoto, Kazunori Kashiwase, Tomokazu Motomura, Yasuhiro Kuroda, Yuji Yasuga, Naohiro Yonemoto, Hiroyuki Yokoyama, Ken Nagao, Hiroshi Nonogi, on behalf of the J-PULSE-Hypo investigators

**Affiliations:** Emergency and General Medicine, Kumamoto University Hospital, 1-1-1 Honjo, Chuo-ku, Kumamoto 860-8556 Japan; Advanced Medical Emergency and Critical Care Center, Yamaguchi University Hospital, Ube, Japan; Senri Critical Care Medical Center, Saiseikai Senri Hospital, Osaka, Japan; Division of Cardiovascular Care Unit, Department of Cardiovascular Medicine, National Cerebral and Cardiovascular Center, Suita, Japan; Department of Emergency Medicine, Sapporo Medical University School of Medicine, Sapporo, Japan; Department of Cardiology, Hiroshima City Hospital, Hiroshima, Japan; Division of Cardiology, Kokura Memorial Hospital, Kokura, Japan; Emergency Medicine, Osaka Mishima Emergency and Critical Care Center, Osaka, Japan; Emergency and Critical Care Medical Center, Osaka City General Hospital, Osaka, Japan; Cardiovascular Division, Osaka Police Hospital, Osaka, Japan; Department of Emergency and Critical Care Medicine, Chiba Hokusoh Hospital, Nippon Medical School, Inzai, Japan; Emergency and Critical Care Center, Kagawa University Hospital, Kagawa, Japan; Department of Cardiology, Sumitomo Hospital, Osaka, Japan; Department of Psychopharmacology, National Center of Neurology and Psychiatry, Tokyo, Japan; Department of Cardiology, Cardiopulmonary Resuscitation and Emergency Cardiovascular Care, Surugadai Nihon University Hospital, Tokyo, Japan; Shizuoka General Hospital, Shizuoka, Japan

**Keywords:** Target temperature, Rapid cooling, Prolonged hypothermia, Slow rewarming, Post-cardiac arrest syndrome

## Abstract

**Background:**

Therapeutic hypothermia (TH) is a standard strategy to reduce brain damage in post-cardiac arrest syndrome (PCAS) patients. However, it is unknown whether the target temperature should be adjusted for PCAS patients in different states.

**Methods:**

Participants in the J-PULSE-Hypo study database were divided into lower (32.0–33.5 °C; Group L) or moderate (34.0–35.0 °C; Group M) temperature groups. Primary outcome was a favourable neurological outcome (proportion of patients with a Glasgow-Pittsburgh Cerebral Performance Category [CPC] of 1–2 on day 30). We compared between the two groups and in subgroups of patients divided by age and resuscitation interval (interval from collapse to return of spontaneous circulation) by propensity score (PS) analysis.

**Results:**

Overall, 467 participants were analysed. The proportions of patients with favourable neurological outcomes were as follows (Group L vs. Group M) (OR; Odds ratio): all patients, 64 % (*n* = 42) vs. 55 % ((*n* = 424) (PS; OR 1.381 (0.596–3.197)), *P* = 0.452) and resuscitation interval ≤ 30 min, 88 % (*n* = 24) vs. 64 % ((*n* = 281) (PS; OR 7.438 (1.769–31.272)), *P* = 0.007).

**Conclusions:**

PCAS patients with a resuscitation interval of <30 min may be candidates for TH with a target temperature of <34 °C.

**Trial registration:**

University Hospital Medical Information Network (UMIN) Clinical Trials Registry UMIN000001935; available at: https://upload.umin.ac.jp/cgi-open-bin/ctr/ctr.cgi?function=brows&action=brows&type=summary&recptno=R000002348&language=J.

## Background

The efficacy of therapeutic hypothermia (TH) in post-cardiac arrest syndrome (PCAS) patients was demonstrated by two, randomised controlled studies [1, 2]. Current guidelines for resuscitation now recommend TH of 32–34 °C for 12–24 h in PCAS patients. However, a recent randomised controlled trial, the target temperature management (TTM) study [3], did not show any benefit of TH (33 °C) for PCAS patients when comparing TH (33 °C) and controlled normothermia (36 °C). Therefore, there is still no clear evidence to show whether lower temperature treatment or anti-fever treatment should be considered for managing PCAS patients. Despite the limited clinical evidence, recent animal studies have shown beneficial effects of TH on neurological outcomes [4–6]. This raises the question of what causes the differences in the neurological outcomes between clinical and animal studies. Therefore, we hypothesised that there are some subgroups of patients in clinical settings who may show the greatest efficacy of lower target temperature TH.

The J-PULSE-Hypo study registry is a Japanese prospective cohort observational study of TH in PCAS patients. A total of 14 medical centres participated in this registry, which was originally conducted between January 2005 and 2009, and was then extended to March 2013 [7]. In the present study, we performed analyses of the J-PULSE-Hypo registry database to address our objective, which was to identify subgroups of patients who might be suitable candidates for lower target temperature TH.

## Methods

### Study design

The present study used the J-PULSE-Hypo registry database, a prospective cohort database of PCAS patients who received TH at 14 Japanese hospitals. Patients were registered between January 2005 and December 2012. The study was conducted in accordance with the ethical guidelines for epidemiological studies and was approved by the ethics committee of the National Cerebral and Cardiovascular Center. Ethical review boards at all 14 participating centres approved the study protocol.

### Patients

Patients satisfying the following criteria were eligible for registration: (1) all patients who received TH at any of the 14 participating facilities with successful return of spontaneous circulation (ROSC) after out-of-hospital cardiac arrest; (2) patients aged ≥18 years; (3) stable hemodynamics after ROSC, including stabilisation by drugs or assisted circulation, such as intra-aortic balloon pumping or percutaneous cardiopulmonary support; (4) persistent coma (Japan coma scale score of 200 or 300, or Glasgow coma scale score of 3–5) after ROSC; and (5) presumed cardiac aetiology of cardiac arrest according to the Utstein guidelines [8]. Patients were excluded for the following reasons: pregnancy, aortic dissection, pulmonary embolism, drug addiction, and poor daily activity before the onset of cardiac arrest.

### Treatment

TH was performed with sedation and analgesia according to the hospital’s established procedures. In some cases, ice-cold intravenous fluid was administered over 30–60 min to initiate hypothermia. Hypothermia was initiated and/or maintained by (1) surface cooling with a cooling blanket (Blanketrol II; CSZ Medical, Cincinnati, OH, USA) or a cooling device with self-adhesive, hydrogel-coated pads (Arctic Sun; Medivance, Louisville, KY, USA), or by (2) blood cooling with an extracorporeal direct blood cooling device (KTEK-III, Kawasumi, Tokyo, Japan) or an endovascular cooling device (CoolGard 3000; Alsius, Irvine, CA, USA). Mild hypothermia (32–35 °C) was maintained for 24–72 h. Rewarming was conducted slowly and gradually over at least 24–72 h. The hypothermia protocol was conducted according to each institution’s protocols.

### Study outcomes and statistical analysis

The patients were divided into two groups according to the target temperature as either <34 °C (32.0–33.5 °C; low temperature, Group L) or ≥34 °C (34.0–35.0 °C; moderate temperature, Group M).

The patient’s neurological outcome was assessed using the Glasgow-Pittsburgh Cerebral Performance Category (CPC), which included the following five categories: CPC 1 (good recovery), CPC 2 (moderate disability), CPC 3 (severe disability), CPC 4 (vegetative state), and CPC 5 (death) [9]. In the present study, a favourable primary outcome was defined as CPC 1–2 on day 30.

About cooling method, the duration of time at the target temperature is the duration (h) of keeping the target temperature; rewarming time is the duration (days) with which rewarming starts to reach 36.0 °C; Over-cooling is defined as less than 0.5 °C from target temperature.

The proportion of patients with a favourable neurological outcome was compared between the two groups for all patients and for specified subgroups of patients using univariate and multivariate analyses with propensity score analysis. In the present study, age and resuscitation interval were eligible factors for the subgroup, which were thought of as global factors having influence for the outcome; 60 years old and 30 min were treated as cut-off values from the median of the previous cohort study [10]. The following subgroups of patients were analysed: age ≤60 vs. >60 years and resuscitation interval (interval from collapse to ROSC) of ≤30 vs. >30 min.

Univariate analyses were performed using the Mann-Whitney *U* test and Fisher’s exact test as appropriate. Propensity score analysis was performed by taking into account age, sex (male), bystander cardiopulmonary resuscitation (CPR), cardiogenic cardiac arrest, and resuscitation interval using the inverse probability of the treatment-weighting (IPTW) method. All statistical analyses were considered significant at *P* < 0.05.

Statistical analyses were performed using SPSS software version 16.0 (IBM, Armonk, NY, USA) for all analyses, except for propensity score analysis, which was conducted using R software version 3.1.2 (GNU general public licence).

## Results

The registry included 467 patients, but the target temperature was not stated for ten patients. The characteristics of the analysed patients are summarised in Table 1. Age, target temperature, maintenance of the target temperature, and the rewarming process were significantly different between Groups L and M.

**Table 1:** Patient characteristics

Variable	Group L	Group M	*P* value
	(32.0–33.5 °C)	(34.0–35.0 °C)	
	*n* = 42	*n* = 425	
Age (years)	52 (44–61)	61 (53–69)	<0.001
Male (%)	37 (88 %)	349 (82 %)	0.399
Bystander CPR (%)	18 (43 %)	225 (53 %)	0.257
Witness (%)	27/34 (79 %)	298/373 (80 %)	1.000
Cardiogenic cardiac arrest (%)	41 (98 %)	398 (94 %)	0.497
VF/VT (%)	35/42 (83 %)	286/419 (68 %)	0.052
Target temperature (°C)	33.0 (33.0–33.0)	34.0 (34.0–34.0)	<0.001
Over-cooling (%)	15/42 (36 %)	86/404 (21 %)	0.051
Interval from collapse to ROSC (min)	24 (15–41)	21 (13–34)	0.154
Interval from ROSC to target temperature (min)	180 (105–290)	170 (74–335)	0.657
Duration of time at the target temperature (h)	34 (24–50)	25 (24–38)	0.004
Rewarming time (days)	3 (2–4)	2 (1–3)	<0.001
Cooling method			
Initial infusion of cold fluid	27/42 (64 %)	229/425 (54 %)	0.255
Surface cooling	27/42 (64 %)	197/416 (47 %)	0.051
Device used			
Percutaneous cardiopulmonary support	6/30 (20 %)	84/325 (26 %)	0.661
CPC 1–2 on day 30	27 (64 %)	232 (55 %)	0.258

Values are presented as the *n* (%) or median (interquartile range)

*CPR* cardiopulmonary resuscitation, *VF* ventricular fibrillation, *VT*, ventricular tachycardia, *ROSC* return of spontaneous circulation, *CPC* cerebral performance category

Table 2 compares the proportions of patients with a favourable neurological outcome between Groups L and M for all patients and in each subgroup. The proportion of patients with a favourable outcome was significantly different between Groups L and M in the subgroup of patients with a resuscitation interval of ≤30 min (88 % (*n* = 24) vs. 64 % (*n* = 281) odds ratio (OR) 3.867 (1.126–13.286), *P* = 0.024) [OR; odds ratio (95 % confidence interval)].

**Table 2:** Proportions of patients with favourable neurological outcomes according to the target temperature of therapeutic hypothermia

Variable	Target temperature	*n*	CPC 1–2 on day 30	OR (95 % CI)	*P* value
All patients	<34 °C	42	27 (64 %)	1.490 (0.770–2.881)	0.258
	≥34 °C	424	232 (55 %)		
Age					
≤60 years	<34 °C	30	21 (70 %)	1.167 (0.507–2.685)	0.836
	≥34 °C	204	136 (67 %)		
>60 years	<34 °C	12	6 (50 %)	1.292 (0.404–4.131)	0.769
	≥34 °C	220	96 (44 %)		
Resuscitation interval					
≤30 min	<34 °C	24	21 (88 %)	3.867 (1.126–13.286)	0.024
	≥34 °C	281	181 (64 %)		
>30 min	<34 °C	14	3 (21 %)	0.678 (0.178–2.586)	0.756
	≥34 °C	115	33 (29 %)		

Values are presented as the *n* (%)

*CPC* cerebral performance category, *OR* odds ratio, *CI* confidence interval

Table 3 shows the characteristics of patients in Groups L and M in the subgroup of patients with a resuscitation interval of ≤30 min. The age in the background showed significant difference in this subgroup (50 vs. 62 years old).

**Table 3:** Characteristics of patients with a resuscitation interval of ≤30 min

Variables	Group L	Group M	*P* value
	(32.0–33.5 °C)	(34.0–35.0 °C)	
	*n* = 24	*n* = 281	
Age (years)	50 (38–57)	62 (53–69)	<0.001
Male (%)	19 (79 %)	228 (81 %)	0.789
Bystander CPR (%)	9 (38 %)	146 (52 %)	0.205
Witness (%)	17/21 (81 %)	205/256 (80 %)	
Cardiogenic cardiac arrest (%)	23 (96 %)	263 (70 %)	1.000
VF/VT (%)	21/24 (88 %)	185/277 (67 %)	
Target temperature (°C)	33.0 (32.0–33.0)	34.0 (34.0–34.0)	<0.001
Over-cooling (%)	7/24 (29 %)	54/270 (20 %)	
Interval from collapse to ROSC (min)	17 (11–23)	15 (10–22)	0.469
Interval from ROSC to target temperature (min)	194 (165–324)	180 (95–360)	0.460
Duration of time at the target temperature (h)	34 (24–49)	24 (24–38)	0.013
Rewarming time (days)	3 (2–4)	2 (1–3)	<0.001
CPC 1-2 on day 30 (%)	21 (88 %)	181 (64 %)	0.024

Values are presented as the *n* (%) or median (interquartile range)

*CPR* cardiopulmonary resuscitation, *VF* ventricular fibrillation, *VT* ventricular tachycardia, *ROSC* return of spontaneous circulation, *CPC* cerebral performance category

Table 4 shows the results of the propensity score analysis comparing the proportion of patients with a favourable neurological outcome between Groups L and M. This analyses shows that the proportion of patients with a favourable neurological outcome was significantly different between Groups L and M in the subgroup of patients with a resuscitation interval of ≤30 min (OR; 7.438 (1.769–31.272), *P* = 0.007).

**Table 4:** Comparison of the proportion of patients with a favourable neurological outcome between the two target temperature groups using propensity score analysis with the inverse probability of the treatment-weighting method

Variable	Target temperature	OR (95 % CI)	*P* value
All patients	<34 °C (vs. ≥34 °C)	1.381 (0.596–3.197)	0.452
Age			
≤60 years	<34 °C (vs. ≥34 °C)	0.779 (0.297–2.147)	0.657
>60 years	<34 °C (vs. ≥34 °C)	3.812 (0.945–15.367)	0.061
Resuscitation interval			
≤30 min	<34 °C (vs. ≥34 °C)	7.438 (1.769–31.272)	0.007
>30 min	<34 °C (vs. ≥34 °C)	0.583 (0.145–2.344)	0.449

The propensity score analysis incorporated the following variables: age, sex (male), bystander cardiopulmonary resuscitation, cardiogenic cardiac arrest, and resuscitation interval

*OR* odds ratio, *CI* confidence interval

Table 5 compares the proportion of patients with a favourable neurological outcome in the subgroup of patients with a resuscitation interval of ≤30 min. As shown in this table, the proportion of patients was significantly different between Groups L and M for the overall subgroup and for patients with ventricular fibrillation/ventricular tachycardia (VF/VT) (88 % (*n* = 24) vs. 64 % (*n* = 281) OR; 3.867 (1.126–13.286), *P* = 0.024 and 95 % (*n* = 21) vs. 71 % (*n* = 185) OR; 8.244 (1.079–62.975), *P* = 0.017, respectively).

**Table 5:** Proportions of patients with favourable neurological outcomes according to the target temperature of therapeutic hypothermia in patients with a resuscitation interval of ≤30 min

Variable	Target temperature	*n*	CPC 1–2 on day 30	OR (95 % CI)	*P* value
Resuscitation interval ≤30 min	<34 °C	24	21 (88 %)	3.867 (1.126–13.286)	0.024
	≥34 °C	281	181 (64 %)		
	<34 °C		20 (95 %)	8.244 (1.079–62.975)	0.017
VF/VT	≥34 °C	21	131 (71 %)		
	<34 °C	185	1 (33 %)	0.479 (0.042–5.465)	0.617
Non-VF/VT	≥34 °C	3	47 (51 %)		
		92			

Values are presented as the *n* (%)

*CPC* cerebral performance category, *VF* ventricular fibrillation, *VT* ventricular tachycardia, *OR* odds ratio, *CI* confidence interval

Table 6 shows the results of the propensity score analysis to compare the proportion of patients with a favourable neurological outcome between Groups L and M in a subgroup of patients with an interval of 30 min from collapse to ROSC and VF/VT. The difference in the proportion of patients with a favourable neurological outcome was statistically significant between Groups L and M in this subgroup of patients (OR; 19.43 (2.39–158.04), *P* = 0.006).

**Table 6:** Comparison of the proportion of patients with a favourable neurological outcome between the two target temperature groups using propensity score analysis with the inverse probability of the following treatment-weighting method: patients with a resuscitation interval of ≤30 min and ventricular fibrillation/ventricular tachycardia

Variable	Target temperature	OR (95 % CI)	*P* value
VF/VT	<34 °C (vs. ≥34 °C)	19.43 (2.39–158.04)	0.006

The propensity score analysis incorporated the following variables: age, sex (male), bystander CPR, and resuscitation interval

*OR* odds ratio, *CI* confidence interval

## Discussion

In the present study, TH with a lower target temperature of 32.0–33.5 °C (i.e. Group L) significantly improved the proportion of patients with favourable neurological outcomes in the subgroup of patients with a resuscitation interval of ≤30 min, as compared with TH with a higher target temperature of 34.0–35.0 °C (i.e. Group M) (Table 2). However, it must be acknowledged that with the background characteristics of Groups L and M, the age in the background showed significant difference in this subgroup (50 vs. 62 years old) (Table 3). Therefore, propensity score analysis with the IPTW method was applied, and confirmed the significant difference in the outcome between Groups L and M in this subgroup of patients (Table 4).

We also found a significant difference in the VF/VT ratio between Group L and M (88 % vs. 67 %) in patients with a resuscitation interval of ≤30 min. Therefore, to confirm that the effectiveness of lower target temperature TH was independent of the VF/VT ratio, we compared the outcomes between VF/VT and non-VF/VT cases, because VF/VT cases tend to have better outcomes than non-VF/VT cases [10]. In VF/VT cases, we found that the lower target temperature TH conferred better neurological outcomes, but there was no difference between the two groups of patients in non-VF/VT cases (Tables 5 and 6).

The TTM study included two groups of patients with target temperatures of 33 and 36 °C. The characteristics of both groups were similar in terms of their mean age (33 vs. 36 °C; 64 vs. 64 years old, respectively), proportion of males (83 vs. 79 %, respectively), proportion of patients receiving bystander CPR (73 vs. 73 %, respectively), proportion of patients with VF/VT (73 vs. 73 %, respectively), and mean interval from collapse to ROSC (25 vs. 25 min, respectively) [3]. Comparing the TTM study and our study, it is apparent that our study population was younger, and bystander CPR was less frequent, but the resuscitation interval was similar.

There were some differences between our study and the TTM study, including shorter time to reach the target temperature (our study vs. TTM study; 180 vs. about 480 min), longer time at the target temperature (34 vs. about 20 h), and longer rewarming period (3 days vs. 9 h). Several animal studies have demonstrated potential beneficial outcomes of rapid cooling [11–13]; prolonged TH may reduce secondary brain damage [14]; and slow rewarming may attenuate traumatic brain injury [15–18]. Therefore, it seems that these TH methods improved the neurological outcomes of PCAS patients.

Therefore, there might be a possibility that the fundamental cooling method (prolonged TH and long rewarming period) had an advantage for outcome. Actually, the favourable outcome of the present study (Group L; 64 % and Group M; 55 %) could be superior to the outcome of TTM study (33 °C group; 46 % and 36 °C group; 48 %). This topic should be discussed in a further study.

Based on the results of animal studies, it is thought that lower target temperature TH could improve the neurological outcomes compared with higher target temperatures [4–6]. However, clinical studies have not yet shown similar effects of lower target temperature TH in humans. In the present study, we found that patients with a resuscitation interval of ≤30 min had better outcomes following lower target temperature TH, and this shorter resuscitation interval might mimic the design of animal studies. Irrespective of the reason, it seems that lower target temperature TH might be advantageous in patients with a shorter resuscitation interval.

Extracorporeal cooling is currently applied in the clinical setting, and it is vital that the target temperature is reached within 20 min after starting cooling [19]. A recent clinical study showed that a combination of TH with rapid cooling and a cardiopulmonary support device improved the neurological outcomes compared with conventional methods, when performed about 60 min after VF/VT cardiac arrest. [20] However, it is not clear whether rapid cooling and/or the support device contributed to the improved outcomes. It is possible that quicker cooling could improve the outcome and expand the induction period of TH. In the present study, in which induction cooling was achieved within 180 min, a significant difference between the two target temperatures was only noted for patients with a resuscitation interval of ≤30 min.

This study has some limitations to mention. First, we performed subgroup analyses of an observational study. Therefore, the results should be confirmed in future studies. Second, several cooling methods were used, but any advantages of the cooling methods could not be shown in this study. Third, the effects of the cooling protocol were not analysed in sufficient detail because of differences in the maintenance and rewarming processes between the two groups of patients. Fourth, neurological outcome was assessed on day 30, which might not be enough observation periods.

## Conclusions

Although lower target temperature TH for PCAS patients has not been established, including indications, methods, and procedures, the present results might indicate that lower target temperature TH could be candidate for patients with a resuscitation interval of ≤30 min. We conclude that these results warrant further studies examining the effects of lower target temperature TH in PCAS patients.

## Abbreviations

CPC: Glasgow-Pittsburgh cerebral performance category
OR: odds ratio
PCAS: post-cardiac arrest syndrome
PS: propensity score
ROSC: return of spontaneous circulation
TH: therapeutic hypothermia
TTM: target temperature management
VF/VT: ventricular fibrillation/ventricular tachycardia
